# Restoration of the Normal Splicing Pattern of the *PLP1* Gene by Means of an Antisense Oligonucleotide Directed against an Exonic Mutation

**DOI:** 10.1371/journal.pone.0073633

**Published:** 2013-09-03

**Authors:** Stefano Regis, Fabio Corsolini, Serena Grossi, Barbara Tappino, David N. Cooper, Mirella Filocamo

**Affiliations:** 1 Centro di Diagnostica Genetica e Biochimica delle Malattie Metaboliche, Istituto G. Gaslini, Genova, Italy; 2 Institute of Medical Genetics, School of Medicine, Cardiff University, Cardiff, United Kingdom; University of Texas MD Anderson Cancer Center, United States of America

## Abstract

An exonic missense mutation, c.436C>G, in the *PLP1* gene of a patient affected by the hypomyelinating leukodystrophy, Pelizaeus–Merzbacher disease, has previously been found to be responsible for the alteration of the canonical alternative splicing profile of the *PLP1* gene leading to the loss of the longer PLP isoform. Here we show that the presence of the c.436C>G mutation served to introduce regulatory motifs that appear to be responsible for the perturbed splicing pattern that led to loss of the major PLP transcript. With the aim of disrupting the interaction between the *PLP1* splicing regulatory motifs and their cognate splicing factors, we designed an antisense oligonucleotide-based *in vitro* correction protocol that successfully restored PLP transcript production in oligodendrocyte precursor cells.

## Introduction

Pelizaeus–Merzbacher disease (PMD, MIM #312080) and X-linked paraplegia type 2 (SPG2, MIM #312920) are allelic hypomyelinating leukodystrophies caused by mutations in the *PLP1* gene (MIM #300401). The resulting clinical phenotypes vary quite widely, ranging from the most severe connatal phenotype, presenting at birth with nystagmus, severe spasticity and hypotonia, to relatively mild cases of paraplegia without mental retardation [1].

Duplication of a region of chromosome Xq22.2 containing the *PLP1* gene is the most frequent gene defect reported in PMD (60-70% of cases). The complete deletion of the gene has been reported only rarely as a cause of PMD/SPG2, while point mutations have been identified in 20% of cases [2]. The *PLP1* gene, which is mainly expressed in oligodendrocytes, encodes a 4-pass transmembrane protein, termed PLP, which is the most abundant protein in the myelin sheaths of the central nervous system (CNS) [3]. A shorter protein isoform, DM20, which lacks 35 amino acids from an intracellular domain, is however generated by alternative splicing of the same primary *PLP1* gene transcript. Two competing 5’ donor splice sites, residing within the 3’ portion of exon 3 (commonly termed exon 3B) are responsible for the alternative splicing [4]. The biological roles of the PLP/DM20 proteins remain to be clarified.

Despite their abundance in CNS myelin, myelination occurs in knockout mice lacking the *Plp1* gene, even though the myelin produced exhibits reduced physical stability [5]. In similar vein, male PMD patients harbouring a complete *PLP1* gene deletion are relatively mildly affected [1]. However, male PMD patients with a duplication of the *PLP1* gene exhibit a more severe phenotype, with severity increasing in the rarer PMD patients with three or five *PLP1* gene copies [6]. Point mutations can give rise to a wide range of PMD/SPG2 phenotypes, the most severe being those that impair PLP and DM20 folding and transport [7], whilst mutations with a limited impact on PLP/DM20 protein structure are predicted to lead to milder phenotypes. Among these mild mutations are those that are located in exon 3B and which therefore involve PLP but not DM20 [3].

We previously reported a mildly affected PMD patient with a c.436C>G mutation located in exon 3B, and demonstrated that this mutation, which would be expected to substitute leucine at residue 146 with a valine (p.L146V), resulted instead in the loss of the PLP isoform [8]. Although none of the regulatory splice sites known to be involved in PLP/DM20 alternative splicing regulation [9–11] was directly altered by the mutation, computational analysis suggested that c.436C>G leads to the acquisition of exon splicing silencer (ESS) motifs [8]. In the present report, we have extended this analysis by identifying several splicing regulatory motifs created by the c.436C>G mutation. *In vitro* experimentation subsequently confirmed the predictions of the *in silico* hypotheses and suggested the therapeutic potential of a specific antisense oligonucleotide to correct the splicing defect by blocking the splicing regulatory motifs.

## Materials and Methods

### Plasmid constructs, transfection and transcript analysis

Plasmid constructs, derived from the pcDNA3.1/V5-His-TOPO/LacZ vector (Invitrogen, San Diego, CA), with a recombinant in-frame LacZ-PLP1-LacZ minigene containing the genomic region between exons 2 and 4 of the *PLP1* gene, were previously prepared as wild-type and mutant (c.436C>G) versions [8]. In the present study, a double mutant construct was produced using the QuikChange II Site-Directed Mutagenesis Kit (Stratagene Agilent, Santa Clara, CA, USA). In accordance with the manufacturer’s instructions, specific primers (146UPF: 5’-GTTTGGGAAAATGGGAAGGACATCCCGACAAG-3’ and 146UPR: 5’-CTTGTCGGGATGTCCTTCCCATTTTCCCAAAC-3’) were used to introduce a further mutation (c.437T>A) adjacent to the c.436C>G in the mutant plasmid. Thus, three versions of the construct were used: the wild-type version (TGGCTAGG), the naturally occurring mutant version (TGGGTAGG), and the double mutant version (TGGGAAGG).

Wild-type and mutant plasmid constructs were transfected into murine oligodendroglial Oli-neu cells [12] using the Lipofectamine 2000 Transfection Reagent (Invitrogen) according to the manufacturer’s instructions. SRSF6-specific siRNA (Ambion, Austin, TX, USA) was cotransfected with the mutant (c.436G) minigene into Oli-neu cells using the lipofectamine 2000 Transfection Reagent (Invitrogen) according to the manufacturer’s guidelines. A morpholino antisense oligonucleotide (Gene Tools, Philomath, OR, USA) (5’-GATGTCCTACCCATTTTCCCAAACA-3’) was designed by the Gene Tools Oligo Design Support based upon the target sequence surrounding the c.436G mutation. When morpholino treatment was performed, various amounts of morpholino oligonucleotide were added with the EndoPorter peptide delivery system (Gene Tools) to the c.436G mutant minigene-transfected Oli-neu cells as detailed in the Results and Discussion section.

Transfected cells were harvested 48 hrs after transfection. RNA was extracted using an RNeasy Plus Mini kit (Qiagen, Valencia, CA, USA) and reverse transcribed using the SuperScript® VILO™ cDNA Synthesis Kit (Invitrogen). To avoid reverse transcription of endogenous *Plp1* gene transcripts from the Oli-neu cells, when specific priming was required, first-strand cDNA synthesis was performed using the SuperScript III First-Strand Synthesis System for RT-PCR (Invitrogen), using a minigene-specific primer, LACT2R: 5’- CGCGGGCCCTCTAGACTCGA -3’, according to Grossi et al. [8]. RT-PCR from non-specifically primed reverse transcription reactions was performed using both minigene-specific primers 31GF: 5’- TGATTTCAGCCGCGCTGTACTGG-3’ and LACT2R [8].

To evaluate PLP transcript expression in morpholino-treated Oli-neu cells transfected with the mutant plasmid construct, we performed real-time PCR on cDNA reverse transcribed from LACT2R-primed RNA using a PLP transcript-specific primer-TaqMan probe set encompassing *PLP1* exons 3 and 4 (P2), and a PLP/DM20 transcripts-specific primer-TaqMan probe set encompassing *PLP1* exons 2 and 3 (P23B). Set P2 was used as target, whereas set P23B was used as a reference [8]. Using this experimental approach, we were able to derive the PLP/(DM20+PLP) ratio for cells treated with the morpholino antisense oligonucleotide. cDNA samples, reverse transcribed from LACT2R-primed RNAs, were run in quadruplicate, each well containing the cDNA obtained from 10 ng RNA. A plasmid containing the cDNA corresponding to PLP transcript isoform was used to generate the standard curves for the P2 and the P23B amplicons, respectively. Standards were run in duplicate. Standard wells contained 10^-2^, 10^-3^, 10^-4^, 10^-5^, 10^-6^ and 10^-7^ ng/µl plasmid DNA.

### Patient samples

All patient samples were obtained from the “Cell Line and DNA Biobank from Patients Affected by Genetic Diseases” (G. Gaslini Institute, Genoa) and processed as previously described [8].

### Ethical aspects

Following ethical guidelines, all cell and nucleic acid samples stored in the Biobank were obtained for analysis and storage with the patients’ (and/or a family member’s) written informed consent. Consent was sought using a form approved by local Ethics Committee.

## Results and Discussion

In a previously studied PMD patient, we identified a *PLP1* gene mutation, c.436C>G, located within exon 3B, the PLP-specific region [8]. Since no RNA sample was available from the patient, the functional effect of the c.436C>G mutation was investigated using a recombinant LacZ-PLP1-LacZ in-frame minigene containing a *PLP1* gene exon 2 – exon 4 fragment cloned into the pcDNA3.1/V5-His-TOPO/LacZ vector. Comparison of the mRNAs generated through the transfection of the wild-type (c.436C) and mutated (c.436G) versions of the construct into Oli-neu cells, demonstrated that the c.436C>G, expected to result in a missense p.L146V mutation, led instead to the loss of the PLP transcript encoding the major isoform of the *PLP1* gene, whereas the shorter DM20 version of the *PLP1* transcript was produced normally [8].

**Figure 1:**
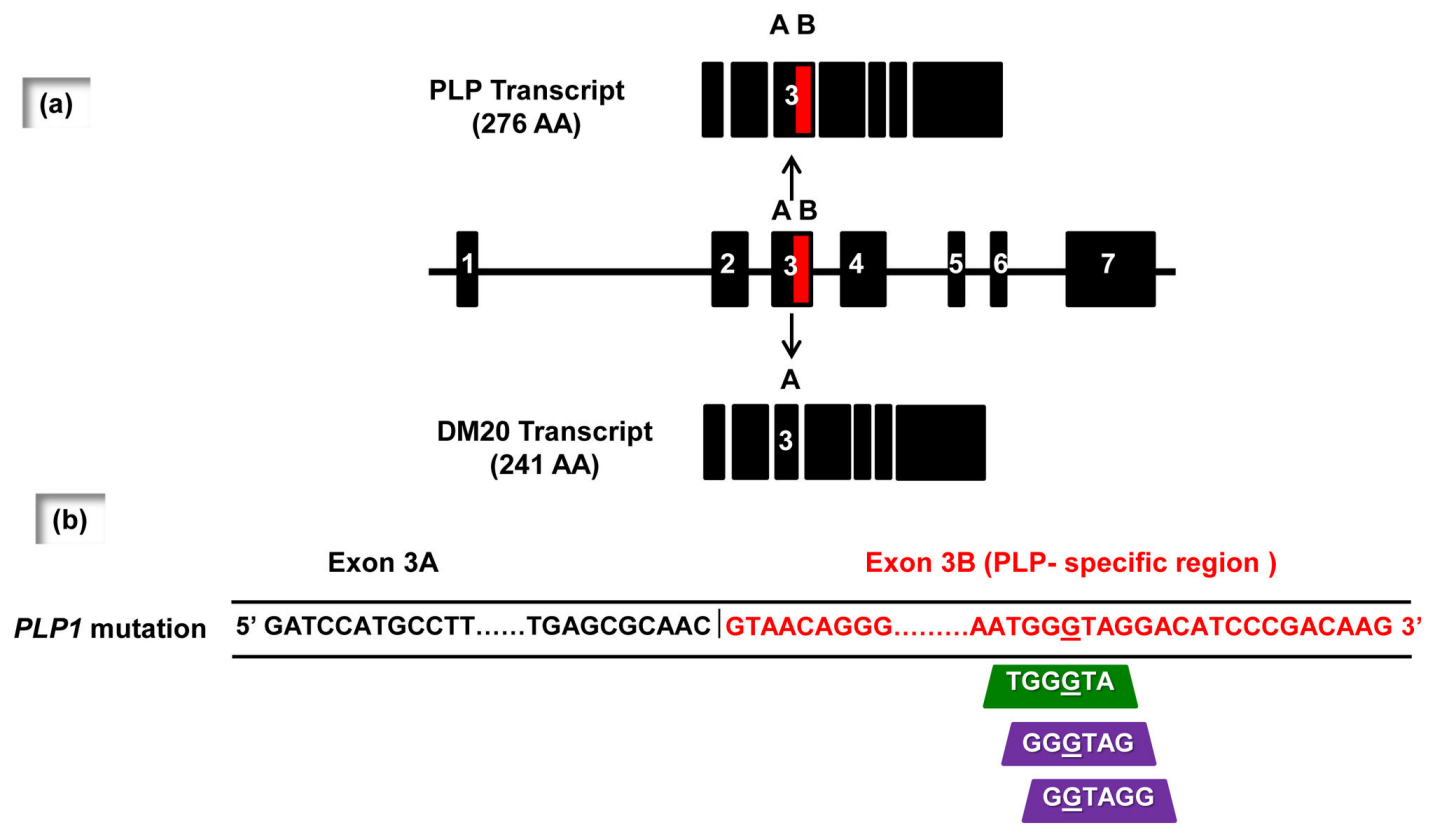
Schematic representation of the analysis of *PLP1* gene structure with the ESEfinder, Human Splicing Finder and FAS-ESS programs. (a) Schematic representation of *PLP1* gene structure (7 exons) and the alternative splicing of the *PLP1* gene into the major *PLP1* transcript and a minor transcript encoding the shorter DM20 isoform, which differs only in terms of the latter half of exon 3 (3B) which is post-transcriptionally spliced out. (b) Only part of the sequence of exon 3A (sequence in black type) and exon 3B (sequence in red type) mutant *PLP1* transcript is represented. The mutated nucleotide (c.436C>G) is underlined. According to the ESEfinder 3.0 program, the c.436C>G change created an exonic splicing enhancer (EES, green trapezium), whereas Human Splicing Finder version 2.4.1 and FAS-ESS web server predicted the creation of two exonic splicing silencers (ESS, purple trapezia).

To investigate the apparent association between the c.436C>G mutation and the observed alteration in the mRNA splicing phenotype, we analyzed the mutated region *in silico* to ascertain whether the mutation was responsible for the gain or loss of splicing regulatory motifs. The ESEfinder 3.0 program [13,14] predicted that the c.436C>G mutation would create an exonic splice enhancer (ESE, sequence TGGGTA, mutation site underlined) specific for the SRSF6 splicing factor with a score (2.70848) that was above the threshold level (2.676); the corresponding score for the wild-type sequence (TGGCTA, score: 0.40451) was considerably lower (Table 1 and Figure 1). SRSF6 (also known as Srp55) is a splicing factor belonging to the SR protein family which, by binding to ESEs, helps to ensure the correct 5’ to 3’ linear order of exons in spliced mRNA and also prevents exon skipping [15]. It is therefore likely that SRSF6, by acting negatively on the splicing of exon 3B, plays a role in promoting PLP transcript production. The Human Splicing Finder program (version 2.4.1) [16] and the FAS-ESS web server [17] predicted the creation of two exonic splice silencers (ESS), more specifically two FAS-hex3 hexamers (GGGTAG and GGTAGG) [16] (Figure 1). FAS-hex3 hexamers are potential exonic splicing silencer motifs [17]. They were found to be contained (and were overrepresented) in decamers selected from a decamer random library by a cell-based splicing reporter system specifically designed to detect sequences with splicing silencer activity [17]. Fas-hex3 hexamers could therefore be responsible for the loss of the PLP transcript isoform.

**Table 1:** Output of ESEfinder program when the wild-type and mutated *PLP1* exon 3B sequences are compared.

**Sequence**	**Splicing factor motif**	**Score**	**Threshold score**
TGGCTA (Wt)	SRSF6	0.40451	2.676
TGGGTA (M)		2.70848	
TGGG AA (DM)		0.10328	

Legend: the mutated nucleotides are underlined; Wt = wild-type; M = mutant; DM = double mutant.

**Figure 2:**
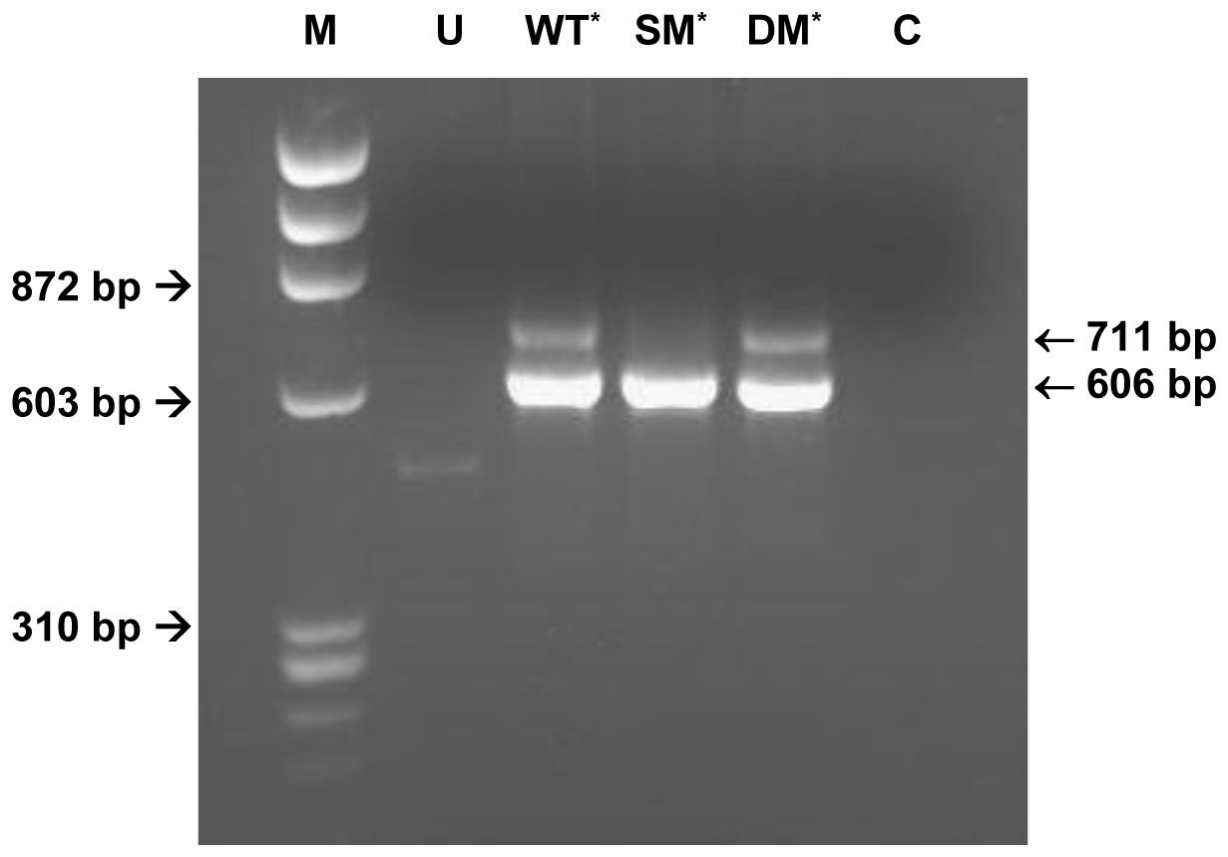
RT-PCR from Oli-neu cells transfected with the wild-type and mutated minigenes. Lane U: untransfected cells; lane WT: cells transfected with the wild-type minigene construct; lane SM: cells transfected with the minigene construct harbouring the c.436C>G mutation; lane DM: cells transfected with the minigene construct harbouring the double mutation [c.436C>T plus c.437T>A]; lane C: negative (no template) control; lane M: φX174 DNA *Hae*III-restricted molecular weight marker. The minigene-specific primer 31GF/LACT2R-mediated RT-PCR products are 711 bp (PLP product) and 606 bp (DM20 product) in length, respectively. The asterisk denotes samples transfected with the pcDNA3.1/V5-His-TOPO/LacZ-PLP1-LacZ vector.

By means of *in silico* analysis, we determined that the identified regulatory motifs (ESE, TGGGTA and ESS, GGGTAG and GGTAGG), could be functionally altered by introducing a second mutation (c.437T>A) immediately adjacent to c.436C>G. Indeed, the resulting double mutant (TGGG AA, GGG AAG and GG AAGG) not only abolished the FAS-hex3 hexamer ESSs but also led to a significant reduction in the ESE score (Table 1). Thus, the newly introduced c.437T>A mutation would be predicted to abolish the effect of the c.436C>G mutation on splicing thereby potentially restoring PLP transcript production. To experimentally confirm this postulate, we prepared by site-specific mutagenesis a double-mutant version of the recombinant minigene construct and transfected the three versions of the minigene [(wild-type (TGGCTAGG), single mutant (TGGGTAGG) and double-mutant (TGGG AAGG)] into Oli-neu cells. As shown in Figure 2, the PLP transcript, which was present in cells transfected with the wild-type minigene but absent in cells transfected with the single mutant minigene, was expressed in cells transfected with the double-mutant minigene. This served to confirm the direct relationship between the presence of the c.436C>G mutation-introduced regulatory motifs and the loss of the PLP transcript isoform.

Having analyzed separately the potential effects of the ESE and the ESS motifs, we surmise that SRSF6, by binding to the ESE site, would direct the mRNA splicing phenotype towards the PLP isoform. To test this hypothesis experimentally, we cotransfected the mutant c.436C>G minigene construct and an SRSF6-specific siRNA into Oli-neu cells. As predicted, the mRNA splicing pattern, lacking the PLP transcript isoform, was unmodified by the siRNA-mediated inhibition of the SRSF6-ESE interaction (Figure S1). These results were consistent with the hypothesis that the observed impact of the c.436C>G mutation on splicing, leading to the loss of the PLP transcript isoform, was mediated by the ESS motifs. We therefore concluded that it was likely that the two newly created ESS motifs played a role in the loss of the PLP isoform.

**Figure 3:**
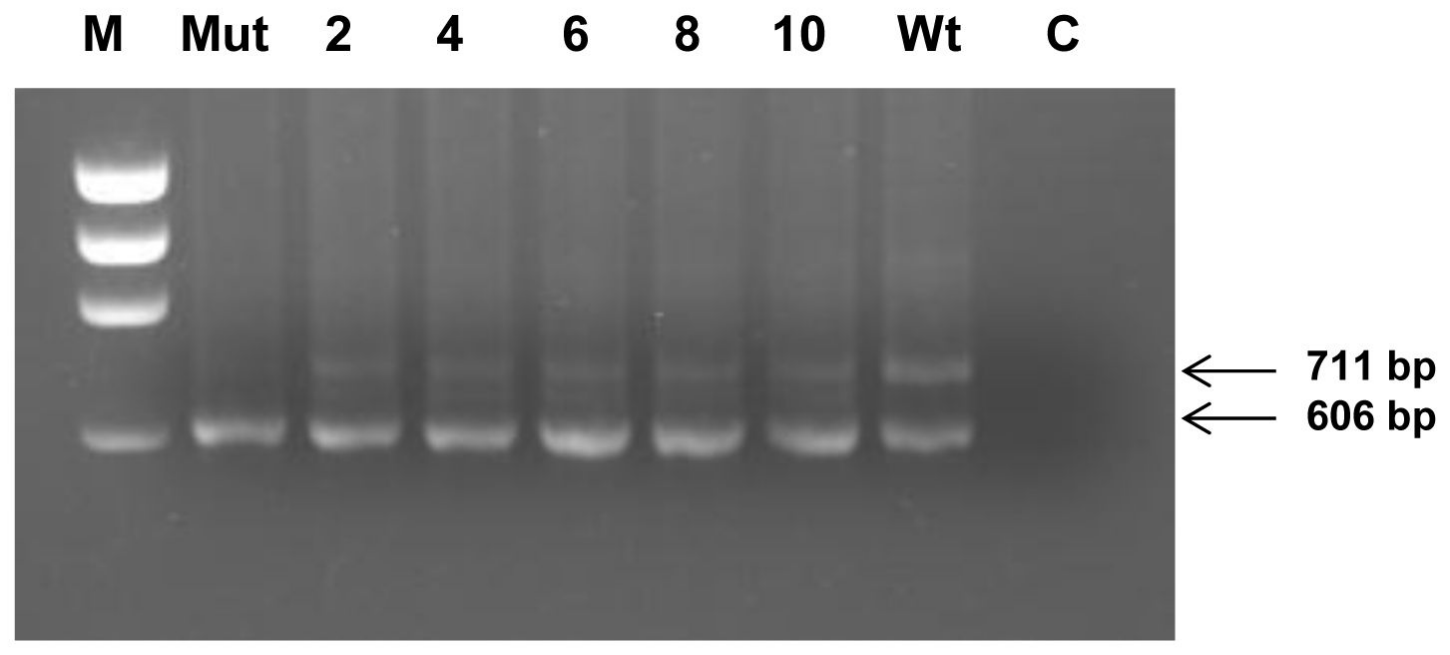
Analysis of morpholino treatment: RT-PCR from minigene-transfected morpholino-treated Oli-neu cells. RNA was extracted from cells transfected with the wild-type (Wt) and mutant (c.436C>G) minigene constructs (Mut, 2, 4, 6, 8, 10). Samples 2, 4, 6, 8 and 10 were treated with the morpholino oligonucleotide, whereas samples Mut and Wt were untreated. Treated cells received 10 mM morpholino oligonucleotide in 2, 4, 6, 8, 10 mM Endoporter reagent; lane C: no template control; lane M: φX174 DNA *Hae*III restricted molecular weight marker. The minigene-specific primer 31GF/LACT2R-mediated RT-PCR products are 711 bp (PLP product) and 606 bp (DM20 product) in length, respectively.

On the basis of this working hypothesis, we next investigated the possibility of correcting *in vitro* the splicing defect caused by the c.436G mutation by preventing the interaction of the identified consensus sequences with their cognate splicing factors. To this end, we employed an antisense oligonucleotide designed to target the mutated region. When the antisense oligonucleotide, specifically a morpholino oligonucleotide, was introduced into Oli-neu cells transfected with the mutant plasmid, restoration of PLP-transcript isoform expression was observed. Although transcript rescue was not effectively achieved using less than 10µm morpholino oligonucleotide (not shown), satisfactory results were obtained with 10µM morpholino delivered with different amounts of Endoporter transfection reagent (from 2 to 10 µM) (Figure 3).

To evaluate quantitatively the morpholino-induced PLP isoform transcript production, we performed real-time PCR experiments using a primer-TaqMan probe-set specific for the PLP transcript (P2) and a primer-TaqMan probe-set specific for both the PLP and DM20 transcripts (P23B), as previously described [8]. Following this procedure, we determined the PLP/(PLP+DM20) ratio for the morpholino-treated Oli-neu cells tranfected with the mutant construct; as shown in Table 2, we obtained PLP/(PLP+DM20) ratios ranging from 0.079 to 0.122. These values compare with a PLP/(PLP+DM20) ratio of 0.212 for Oli-neu cells transfected with the wild-type construct. Thus, employing the morpholino oligonucleotide, we succeeded in increasing the PLP/(DM20+PLP) ratio to a level which was 58% of the wild–type ratio. Although the presence of the PLP and DM20 protein isoforms could not be directly confirmed experimentally, we suspect that the translation of the detected PLP and DM20 transcript might occur without blocking, owing to the mRNA-morpholino interactions, as the region involved is largely downstream of the ATG (approx. 1.9kb) translational start site in the LacZ-PLP1-LacZ minigene. It is already known that the blocking efficiency of morpholino oligonucleotides falls dramatically as the distance of the binding region from the initial ATG increases [18]. It should be noted that these results were obtained using Oli-neu cells as recipients in all the transfection experiments. The use of these mouse oligodendrocyte precursor cells was deemed appropriate with a view to mimicking the cellular environment in which PMD displays its effects, and also avoiding potential biases in splicing selection due to the action of tissue-specific factors.

**Table 2:** Real-time RT-PCR analysis of morpholino-treated Oli-neu cells transfected with mutated (Mut) and wild-type (Wt) *PLP1* minigene constructs.

	**Mut**	**2**	**4**	**6**	**8**	**10**	**Wt**
**PLP/(PLP+DM20) ratio**	0.002	0.122	0.094	0.079	0.094	0.116	0.212
**REC %**	0.9	58	44	37	44	55	100

Legend: Columns 2, 4, 6, 8 and 10 indicate samples treated with 10 µM morpholino oligonucleotide in 2, 4, 6, 8 and 10 µM Endoporter reagent, respectively. The PLP/(PLP+DM20) ratio, obtained using the P2 and P23B sets of primers and probes, is reported for each sample. Rec % denotes the proportion of recovered *PLP1* transcript expression as compared to the wild-type.

The successful oligonucleotide-mediated restoration of the major PLP transcript expression is important because the ultimate cause of the disease in this patient is the absence of the PLP isoform. However, unlike the situation with the DM20 transcript, restoration of the PLP transcript would be expected to lead to the production of a PLP protein isoform harbouring the p.L146V substitution. We neither know, nor can we predict, the likely clinical severity associated with the hypothetical PLP p.L146V mutation as compared with the complete absence of the PLP isoform. The p.L146V mutation would be located within the cytoplasmic loop of PLP. The analysis of the extent of evolutionary conservation of this loop, flanking the Leu 146 residue, in 7 orthologous PLP proteins showed that it has been conserved in amphibians but not in fish, suggesting that the PLP isoform is typical of higher vertebrates (Table S1) [19]. In general, mutations located within this loop, and, in particular, mutations located in amino acid residues in the vicinity of Leu146, are not associated with severe clinical phenotypes. Indeed, the closest reported missense mutations, H147Y [20] and H140Y [21], both of which are predicted to lead to the production of a mutant PLP isoform, have been reported in patients affected by a relatively mild SPG2 clinical phenotype.

In addition, mutations residing within the cytoplasmic loop do not appear to exert a particularly detrimental impact on the general structure of the PLP protein, in particular on the tetraspan structure, whose integrity prevents retention of the protein in the endoplasmic reticulum (ER) [22] with consequent potential activation of the unfolded protein response (UPR). The p.L146V mutation would not be predicted to alter any of the previously proposed putative ER-retention signals: namely, the fourth transmembrane domain of the PLP protein [23], and a heptapeptide within the intracellular loop [24]. Finally, the conservative replacement of the aliphatic hydrophobic side chain of Leu with the slightly smaller aliphatic hydrophobic side chain of Val is not inconsistent with a mild clinical phenotype.

Taking the above considerations together, we would therefore predict that the p.L146V mutation should not give rise to a major structural alteration of PLP nor is it likely that it would result in the retention of the PLP protein isoform in the ER. We would therefore predict that the presence of the L146V-mutated PLP isoform would be less detrimental to the individual harbouring it than would the complete absence of the PLP isoform. If this turns out to be the case, then our oligonucleotide-mediated *in vitro* correction strategy should have a high likelihood of being successful in ameliorating the clinical phenotype of the patient as part of a sequence-targeted therapeutic approach in this case of PMD.

## Supporting Information

Figure S1 siRNA-mediated inhibition of the SRSF6-ESE interaction.
(TIF)

Table S1 **Evolutionary comparison of the PLP1 protein intracellular loop flanking the L146V missense mutation with their orthologous counterparts in seven vertebrates.**
(DOCX)
