# Supplementary figures and images for: Restoration of the Normal Splicing Pattern of the PLP1 Gene by Means of an Antisense Oligonucleotide Directed against an Exonic Mutation
Source: PLoS One. 2013 Sep 3;8(9):e73633. doi: 10.1371/journal.pone.0073633 (PMC3760819; doi:10.1371/journal.pone.0073633)

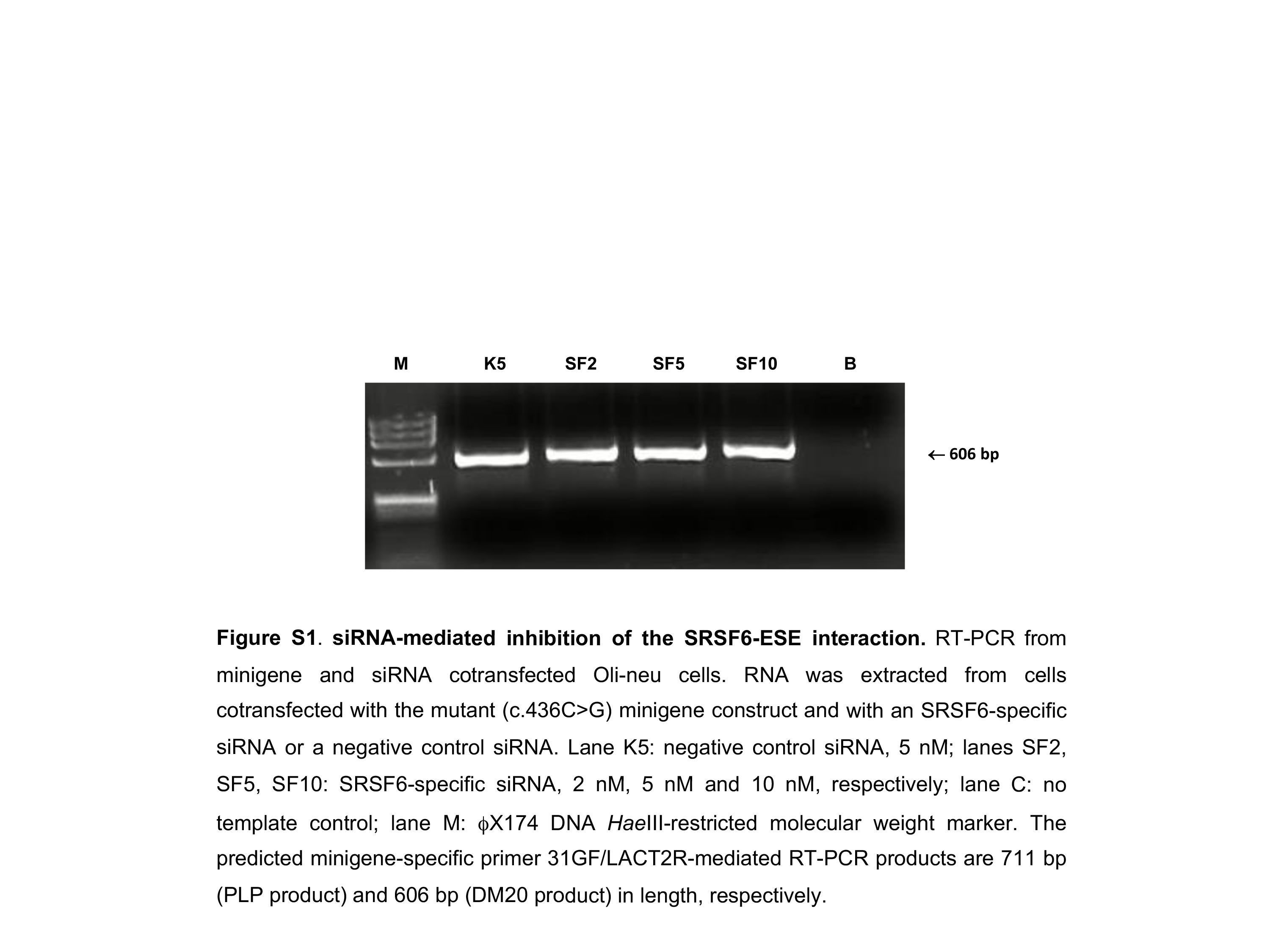

Supplement: Figure S1 — siRNA-mediated inhibition of the SRSF6-ESE interaction. (TIF) [file pone.0073633.s001.tif]
