# Supplementary material for: Restoration of the Normal Splicing Pattern of the PLP1 Gene by Means of an Antisense Oligonucleotide Directed against an Exonic Mutation
Source: PLoS One. 2013 Sep 3;8(9):e73633. doi: 10.1371/journal.pone.0073633 (PMC3760819; doi:10.1371/journal.pone.0073633)
Supplement: Table S1 — Evolutionary comparison of the PLP1 protein intracellular loop flanking the L146V missense mutation with their orthologous counterparts in seven vertebrates. (DOCX) [file pone.0073633.s002.docx]

| **Table S1**. **Evolutionary comparison of the PLP1 protein intracellular loop flanking the L146V missense mutation with their orthologous counterparts in seven vertebrates**. | | | |
| --- | --- | --- | --- |
| NP_000524.3: *Homo sapiens* PLP1 | 89 | EGFYTTGAVRQIFGDYKTTICGKGLSATVTGGQKGRGSRGQHQAHSLERVCHCLGKWLGHPDKFVGITYALTVVWLLVF | 167 |
| NP_955772.1: *Homo sapiens* DM20 | 89 | EGFYTTGAVRQIFGDYKTTICGKGLSAT-----------------------------------FVGITYALTVVWLLVF | 132 |
| XP_003317647.1:Pan troglodytes PLP1 | 89 | EGFYTTGAVRQIFGDYKTTICGKGLSATVTGGQKGRGSRGQHQAHSLERVCHCLGKWLGHPDKFVGITYALTVVWLLVF | 167 |
| NP_035253.1:Mus musculus Plp1 | 89 | EGFYTTGAVRQIFGDYKTTICGKGLSATVTGGQKGRGSRGQHQAHSLERVCHCLGKWLGHPDKFVGITYALTVVWLLVF | 167 |
| NP_112252.1:Rattus norvegicus Plp1 | 89 | EGFYTTGAVRQIFGDYKTTICGKGLSATVTGGQKGRGSRGQHQAHSLERVCHCLGKWLGHPDKFVGITYALTVVWLLVF | 167 |
| NP_990608.1:Gallus gallus Plp1 | 89 | EGFYTTGAVRQIFGDYRTTICGKGLSATVTGGPKGRGARGPQRAHSLQRVCQCLGKWLGHPDKFVGITYVLTIVWLLAF | 167 |
| NP_001082268.1:Xenopus laevis Plp1-a | 89 | EGFYTTTAIKHILGEFKPPAIKGGLISTVTGGTPKGRSTRGRQPVHTIELICRCLGKWLGHPDKFVGVTYIITILWILIF | 168 |
| NP_001079734.1:Xenopus laevis Plp1-b | 89 | EGFYTTTAIKHILGEFKPPAMKGGLISTVTGGPPKGRSTRGRQPVHTIELICRCLGKWLGHPDKFVGVTYVITILWILIF | 168 |
| NP_001027507.1:Xenopus tropicalis Plp1 | 89 | EGFYTTTAIKHILGEFKPPAIKGGLISTVTGGPPKGRSPRGRQPVHTIELICRCLGKWLGHPDKLVGVTYVITILWLLIF | 168 |
| NP_783166.1: Danio rerio Plp1a | 89 | EGFYTTSAVRQSLGEFRSTVCGRCLSTT-----------------------------------FIVITYFLVVVWLLVF | 132 |
| NP_001005586.1: Danio rerio Plp1b | 101 | EGFYTTSAVKQTFGEFRSTQFGRCISLT-----------------------------------FIILTYVLALIWLIVF | 144 |
| Legend: Blue loop is highly conserved being present in mammals, birds, amphibians but not in fish. The Leu residue involved in the L146V change is denoted by a red character. | | | |
